# Does educational mobility in mid-life affect mortality? A cohort study covering 1.3 million individuals in Sweden

**DOI:** 10.1016/j.ssmph.2023.101589

**Published:** 2023-12-23

**Authors:** M. Balaj, H. Sjöqvist, L. van der Velde, PA. Allebeck, AN. Shaaban, S. Swartling Peterson, TA. Eikemo, EE. Agardh

**Affiliations:** a Centre for Global Health Inequalities Research (CHAIN), Department of Sociology and Political Science, Norwegian University of Science and Technology (NTNU), Trondheim, Norway; b Department of Global Public Health, Karolinska Institutet, Stockholm, Sweden

## Abstract

• Studies on how increased formal educational level in mid-life affects mortality is lacking.
• We found that women who increased their educational level in mid-life had a reduced risk of mortality.
• In men, mortality was reduced only for those who increased their education from a low level.

## 1 Introduction

Low educational attainment has been established as a predictor of premature mortality (Balaj et al., 2021; Phelan, Link, Diez-Roux, Kawachi, & Levin, 2004; Ross & Mirowsky, 2010). This effect has been identified as substantial, independent, and consistent across age, sex, and world regions (Baker, Leon, Greenaway, Collins, & Movit, 2011; Balaj et al.).

Education has been identified as a key marker of social position by several observers, and its major impact on mortality has been examined through various pathways. Generally, these pathways originate from initial life conditions, such as cognitive ability in childhood, childhood socio-economic circumstances and health in early life, which affect both health and educational attainment in adulthood. A more direct pathway is the “learned effectiveness” by which education helps people to control their lives, to cope actively and flexibly, and avoid problems (Diderichsen et al., 2012; Mirowsky, 2017). Other pathways recognize the indirect effect of education through the differential access to valued positions leading to procurement of material resources (increased income, property), social recognition, better working conditions and healthier lifestyle practices. These troughresources can create a status shield effect, which significantly reduces health decline and enables the adoption of strategies to improve health (Balaj, 2022). Notably, the positive health returns of education have also been observed to overcome disadvantages experienced in early life (Bonaccio et al., 2018).

A growing number of studies have investigated the effect of education on mortality using changes in national legislations on compulsory schooling, regional differences in compulsory schooling laws or twin studies. Strong associations have been found between extended compulsory schooling and adult mortality in the US (Lleras-Muney, 2005), United Kingdom (Oreopoulos, 2006) and the Netherlands (van Kippersluis, Owen, & van Doorslaer, 2011). Others have found either small or no effects in Europe (Gathmann, Jürges, & Reinhold, 2015; Lager & Jenny, 2012; Meghir, Palme, & Simeonova, 2018) and in the US. (Black et al., 2015) Twin studies have reported similar mixed results (Behrman et al., 2011; Lundborg, Lyttkens, & Nystedt, 2016; Madsen, Nybo Andersen, Christensen, Andersen, & Osler, 2010). These mixed findings may be seen as puzzling as research has demonstrated the causal effect of education on several determinants of health such as on poverty, health behaviours, relationships, and social connections (Galama, Lleras-Muney, & van Kippersluis, 2018; Hofmarcher, 2021). However, twin, and compulsory schooling studies do suffer from several limitations such as power limitations, lack of control for confounders and data quality issues, which may account for the inconsistent results.

In addition, differences between nations in educational systems and later progression through higher education implies that comparisons of long-term effect in studies from different countries should be made with caution. More important for our research question is that virtually all studies on education and long-term health effects are based on attained education or additional years of schooling in the ordinary school system, or early higher education, in younger persons. In this study we are addressing health effects of adult education, i.e., in persons aged 40–50 years.

Generally, adult learning has been promoted in relation to increased labour force participation, earnings, productivity levels and innovation (Darcovich et al., 1997; Ruhose, Thomsen, & Weilage, 2019; Social Affairs and Inclusion, 2011). We have also witnessed an increased research and policy focus on the nonmarket benefits of adult education, such as social capital, social cohesion, and psychological resources (for example self-esteem and self-efficacy) (Feinstein & Hammond, 2004a; Heckman, Humphries, & Veramendi, 2018; Panitsides, 2013; Ward & Edwards, 2002). Thus, and as noted above, far less attention has been paid to the impact of adult education on health. Among the existing studies, most have examined the effect on mental health (Feinstein, 2002; Hammond, 2004), health behaviours (Feinstein & Hammond, 2004b; Zadworna, 2020)^,^ obesity (Feinstein, 2002) and self-reported health (Hammond & Feinstein, 2006; Yamashita, Bardo, Liu, & Won Yo, 2019). With few exceptions such as for obesity (Feinstein, 2002) these studies find that adult learning can benefit behaviours and health for all individuals.

Influenced by the advent of human capital theories in late 60s, adult education became a central part of the Swedish educational and labour market policies (Rubenson, 2001; Wikström, 2006) . Early investment in adult education made Sweden a frontrunner on adult education policy initiatives promoted by the European Union from the early 90s. Equal chances to education were considered an important component of the welfare state, so persons who for various reasons did not complete compulsory school and/or upper-secondary education should be given another possibility. Reasons could be immigration, illness, or childhood conditions not promoting education. It was also in the interest of the industry and employers to have a well-educated labour force, thus encouraging “lifelong learning.” (Rubenson, 2001, pp. 329–338; Wikström, 2006) Access to adult education (municipal adult education, or Komvux) is since then open to virtually anyone who has an interest in completing secondary school and persons attending indeed have a variety of background characteristics (Vuxenutbildningens betydelse i det svenska utbildningssys temet; SCB). Education in Sweden is usually free of charge, and there are possibilities to apply for various types of grants and loans from the central Student Support board (CSN) to cover costs while studying. However, from age 51 the amount you can borrow is decreasing and after 60 years of age access to this kind of loan ceases.

Despite decades of policy and practice attention to adult learning (World Health Organization, 2013), there are to our knowledge, no studies investigating possible health benefits following additional formal education at older ages. To examine these potential benefits, we have used Swedish population health registers linking socioeconomic determinants and mortality through a unique personal identity number. This unique dataset underpins efforts to address the lack of studies focusing on adult formal education, its effect on mortality and their potential causal relationship. The aim of this study is therefore to investigate if increases in the formal education level at the age of 40–50 years is associated with reduced all-cause mortality in Swedish men and women.

## 2 Methods

### 2.1 Study population

**Fig. 1:**
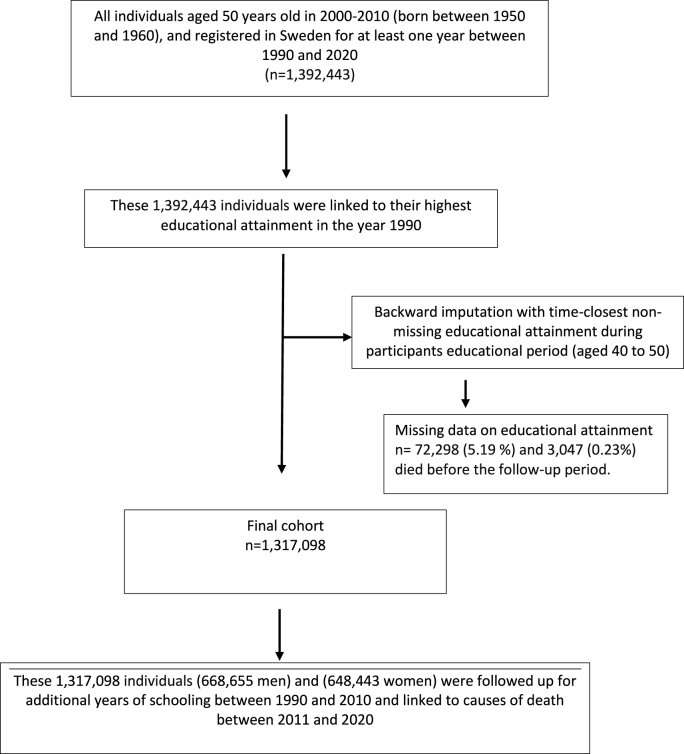
Selection process.

The study population in this retrospective cohort study was selected from a comprehensive register linkage set up for estimating socioeconomic stratification of disease burden. The selection process is shown in Fig. 1 (and Supplementary file Fig. 1). From the Register of the Total Population all individuals aged 50 years old in 2000–2010, e.g., born between January 1st, 1950, and December 31st, 1960, and who had been registered in Sweden for at least one year during January 1st, 1990, to December 31st, 2020, were identified (n = 1,392,443). These individuals were linked by their unique personal identity number to data on their highest attained education in 1990–2000, when they were 40 years old. A unique personal identity number is assigned to each Swedish resident at birth or upon migration to Sweden. They were followed for 10 years to assess additional attained education, when they were between 40 and 50 years of age. We performed a backward imputation with time-closest non-missing educational attainment to fill in potential missing information on highest attained educational attainment during participants educational period (aged 40–50 years). We excluded individuals with no information on education (n = 72,298, 5.2%), and individuals who died before the follow up-period (n = 3,047, 0.23%). The final cohort (n = 1,317,098 individuals; 668,655 men and 648,443 women), was in the final step followed-up for 9 years, between January 1st, 2011, and December 31st, 2020, for death between 51 and 60 years old.

### 2.2 Education

Data on educational attainment was obtained from the Longitudinal Integrated database for Health Insurance and Labour Market Studies (LISA) which comprises information on highest attained education aggregated into the following 7 levels: primary or lower secondary education (less than 9 years), primary or lower secondary education (9 years), upper secondary education (2 years at most), upper secondary education (3 years), post-secondary education (less than 3 years), post-secondary education (3 years or more) and research education. There is also a category for missing information, and those were excluded as described previously.

From these 7 levels, we first created three categories of educational attainment, i.e., low (primary or lower secondary education ≤ 9 years of study), middle (upper secondary education, corresponding to 10–12 years of study) and high (post-secondary or tertiary education, corresponding to >12 years of study). Second, we looked at six groups for analyses: Those with *low*, *middle*, or *high* educational attainment throughout the study period, and those who changed from *low to high*, *low to middle*, and *middle to high*. Even though additional years of schooling can occur horizontally (especially at the post-secondary level, e.g., obtaining another bachelor's or master's degree), in this study we focus on education that leads to upward educational mobility.

### 2.3 All-cause mortality

Deaths were identified through the National Cause of Death Register. This register includes all subjects who died and were registered in Sweden at time of death during one calendar year, regardless of whether death occurred inside or outside the country.

### 2.4 Potential covariates

Potential covariates were selected based on previously documented associations with all-cause mortality; birth year, country of birth, morbidity, unemployment, disposable family income and number of children living at home. Data on unemployment and disposable family income was obtained from LISA, data on other diseases come from the Swedish National in and out- Patient Register and all other covariates were retrieved from the Total Population Register.

### 2.5 Birth year

Year of birth, i.e., born any year between January 1st, 1950, and December 31st, 1960, was treated as a continuous variable.

### 2.6 Country of birth

Country of birth was divided into the following regions: Africa, Asia, EU without Nordics, Europe without EU and Nordics, North America, Nordics without Sweden, Oceania, Russia, Sweden, South America, and unknown and Sweden was the reference group.

### 2.7 Diagnosed morbidity

Through linkage with the national in- and out-patient register, we adjusted for the occurrence of diagnosed morbidity between January 1st in 1990 and December 31st in 2000, when participants were 40 years of age. We selected the diseases with the highest burden in terms of years lived with disability (YLD) in Sweden according to the GBD study. (https://vizhub.healthdata.org/gbd-compare/) These were mental disorders and substance use, musculoskeletal disorders, other non-communicable diseases, neurological disorders, unintentional injuries, skin diseases, chronic respiratory diseases, cardiovascular diseases, and sense organ diseases (Supplementary file Table 1 for ICD-9 and ICD-10 codes). We classified having diagnosed morbidity as yes, if having at least one of the above, and no, if not being hospitalized with any of those disorders between 1990 and 2000.

**Table 1:** Characteristics of men aged 50 years old in 2000–2010 (born between 1950 and 1960), with highest attained education (no change, and change) between the ages 40–50 years (in 1990–2010), and who were followed-up for all-cause mortality between the ages 51–60 (in 2011 and 2020).

Men		Highest attained education between 40 and 50 years of age						
		No change in education			Change in education			
		*Low (*≤ 9 yrs)	*Middle (*10–12 yrs)	*High (*>12 yrs)	*Low to middle*	*Low to high*	*Middle to high*	*p-value
**Total**	*668,655*	146,873 (22)	299,633 (44.8)	195,830 (29.2)	15,275 (2.3)	1837 (0.3)	9207 (1.4)	<0.001
**Died at age 51–60**	Yes	8634 (5.9)	13,133 (4.4)	4762 (2.4)	944 (6.2)	89 (4.8)	314 (3.4)	
	No	138,239 (94.1)	286,500 (95.6)	191,068 (97.6)	14,331 (93.8)	1748 (95.2)	8893 (96.6)	
¥**Diagnosed morbidity at age 40**	No	144,108 (98.1)	295,536 (98.6)	194,503 (99.3)	14,969 (98.0)	1813 (98.7)	9126 (99.1)	<0.001
	Yes	2765 (1.9)	4097 (1.4)	1327 (0.7)	306 (2.0)	24 (1.3)	81 (0.9)	
**Country of birth**	Sweden	120,078 (81.8)	255,033 (85.1)	159,283 (81.3)	10,881 (71.2)	1088 (59.2)	6688 (72.6)	
	Africa	1565 (1.1)	2882 (1.0)	2684 (1.4)	424 (2.8)	95 (5.2)	236 (2.6)	<0.001
	Asia	5828 (4.0)	8449 (2.8)	10,718 (5.5)	1267 (8.3)	271 (14.8)	875 (9.5)	
	EU without Nordics	2854 (1.9)	7272 (2.4)	6385 (3.3)	382 (2.5)	51 (2.8)	319 (3.5)	
	Europe without EU and Nordics	5192 (3.5)	8770 (2.9)	5744 (2.9)	862 (5.6)	158 (8.6)	460 (5.0)	
	North America	267 (0.2)	653 (0.2)	1779 (0.9)	74 (0.5)	32 (1.7)	70 (0.8)	
	Nordics without Sweden	9511 (6.5)	13,339 (4.5)	6487 (3.3)	980 (6.4)	70 (3.8)	312 (3.4)	
	Oceania	46 (<1)	106 (<1)	205 (0.1)	8 (0.1)	2 (0.1)	9 (0.1)	
	Russia	24 (<1)	106 (<1)	275 (0.1)	5 (<1)	10 (0.5)	10 (0.1)	
	South America	1115 (0.8)	2446 (0.8)	1858 (0.9)	300 (2.0)	50 (2.7)	181 (2.0)	
	‡Unknown	393 (0.3)	577 (0.2)	412 (0.2)	92 (0.6)	10 (0.5)	47 (0.5)	
**Unemployment at age 40**	No	100609 (68.5%)	277373 (71.1%)	73783 (69.5%)	8323 (50.5%)	357 (56.7%)	5604 (68.0%)	<0.001
	Yes	11289 (7.7%)	34462 (8.8%)	4722 (4.5%)	4537 (27.5%)	134 (21.3%)	1260 (15.3%)	
	‡Missing	34975 (23.8%)	78486 (20.1%)	27597 (26.0%)	3622 (22.0%)	139 (22.1%)	1383 (16.8%)	
**Family income at age 40**	Low (0–20%)	20,385 (13.9%)	37785 (9.7%)	6036 (5.7%)	3334 (20.2%)	132 (21.0%)	1316 (16.0%)	<0.001
	Lower middle (>20–40%)	26,554 (18.1%)	59,729 (15.3%)	7991 (7.5%)	3300 (20.0%)	147 (23.3%)	1294 (15.7%)	
	Middle (>40–60%)	29,862 (20.3%)	71,862 (18.4%)	13,871 (13.1%)	3631 (22.0%)	114 (18.1%)	1785 (21.6%)	
	Upper middle (>60–80%)	43,956 (29.9%)	121,341 (31.1%)	21,645 (20.4%)	4101 (24.9%)	122 (19.4%)	2098 (25.4%)	
	High (>80–100%)	21,129 (14.4%)	87,542 (22.4%)	47,511 (44.8%)	1447 (8.8%)	52 (8.3%)	1172 (14.2%)	
	‡Missing	4987 (3.4%)	12,062 (3.1%)	9048 (8.5%)	669 (4.1%)	63 (10.0%)	582 (7.1%)	
**Nr. of children at home at age 40**	0	67,854 (46.2)	174,699 (44.8%)	43,902 (41.4%)	8307 (50.4%)	288 (45.7%)	3811 (46.2%)	<0.001
	1	23,934 (16.3)	57892 (14.8%)	1,3045 (12.3%)	2519 (15.3%)	92 (14.6%)	1170 (14.2%)	
	2	41,559 (28.3)	128,207 (32.8%)	3,5725 (33.7%)	3876 (23.5%)	144 (22.9%)	2255 (27.3%)	
	3+	8539 (5.8)	1,7461 (4.5%)	4382 (4.1%)	1111 (6.7%)	43 (6.8%)	429 (5.2%)	
	‡Missing	4987 (3.4)	1,2062 (3.1%)	9048 (8.5%)	669 (4.1%)	63 (10.0%)	582 (7.1%)	

Data are n (%) or p values. *p-values are reported for χ^2^ test. ‡Missing or unknown observations were included in the model as its own categories. ¥Having any of the following at age 40; mental disorders and substance use, musculoskeletal disorders, other non-communicable diseases, neurological disorders, unintentional injuries, skin diseases, chronic respiratory diseases, cardiovascular diseases, and sense organ diseases.

### 2.8 Unemployment

Unemployment is based on the number of days in unemployment as registered with the Swedish Public Employment Agency during one calendar year. We categorized those who had been unemployed ≥90 days as yes, and otherwise no. We measured unemployment during the year the participants were 40 years old.

### 2.9 Disposable family income

Family income is based on the sum of the disposable income of all members of the family divided by the family's total consumption weight. From the distribution of scores among the entire registered population of Sweden, we divided income into five income groups: Low (0–20%), lower middle (>20–40%), middle (>40–60%), upper middle (>60–80%), and high (>80–100%). As for diagnosed morbidity and unemployment we measured the disposaable family income during the year the participants were 40 years old.

### 2.10 Number of children living at home

The number of children living at home was also assessed when participants were 40 years old, e.g., before potential change of education. This selection was made to see whether the number of children could help explain why some people decided to educate themselves further. We categorized number of children living at home into 0, 1, 2 and 3 or more, and did not include their ages.

To adjust for potential unmeasured familial factors from the same upbringing environment, we performed a *sibling analysis*. Examples of shared familial factors include approximately 50% shared genetics, childhood socioeconomic factors, and various lifestyle factors. Those with no siblings, and sibling pairs who were concordant in the outcome, e.g., all-cause mortality during follow-up, were removed from the sibling analysis.

### 2.11 Statistical analyses

For the descriptive statistics, the examinations of potential dependencies between the variables were estimated using Pearson's Chi-squared test. Odds Ratios (ORs) with 95% CIs were estimated in a multiple logistic regression model. We performed analyses on men and women separately. First, we estimated crude ORs for middle and high education and all-cause mortality, as well as changes from low to high and low to middle education, using low education as a reference. In addition, we used the same approach using middle education as a reference. The reason for this was to explore whether higher education matters in relation to those who remained with middle education. Second, we added the covariates one by one in the model, and finally we adjusted for all covariates in the same model simultaneously. Third, we performed a separate sibling analysis, including only those with siblings who were not concordant in the outcome, and we adjusted for all individual covariates simultaneously in this analysis. The sibling analyses were estimated using a fixed effects logistic regression model. By conditioning on the families, we were able to approximately account for all familial factors and 50% of shared genetic variation between the siblings. They were adjusted for all confounders as the previous models, except those who would not vary between siblings. Number of participants in the sibling analysis were 8261 men and 6176 women. We used SAS version 9.4 and Stata/MP 15.1 as software for statistical analysis.

## 3 Results

In men, 29.2 percent had high, 44.8 percent had middle, and 22 percent had low educational attainment at age 40. Approximately 0.3 percent of the men changed their educational attainment from low to high, 2.3 percent from low to middle, and 1.4 percent from middle to high (Table 1). In women, 33.2 percent had high, 44 percent had middle and 15.6 percent low educational attainment. Approximately 0.4 percent of the women changed their education from low to high, 3.6 percent from low to middle, and 3.2 percent from middle to high (Table 2).

**Table 2:** Characteristics of women aged 50 years old in 2000–2010 (born between 1950 and 1960), with highest attained education (no change, and change) between the ages 40–50 years (in 1990–2010), and who were followed-up for all-cause mortality between the ages 51–60 (in 2011 and 2020).

Women		Highest attained education between 40 and 50 years of age						
		No change in education			Change in education			
		*Low (*≤ 9 yrs)	*Middle (*10–12 yrs)	*High (*>12 yrs)	*Low to middle*	*Low to high*	*Middle to high*	*p-value
**Total**	*648,443*	101,291 (15.6)	285,477 (44)	215,556 (33.2)	23,072 (3.6)	2414 (0.4)	20,633 (3.2)	<0.001
**Died at age 51–60**	Yes	4578 (4.5)	8254 (2.9)	4123 (1.9)	748 (3.2)	47 (1.9)	409 (2.0)	
	No	96,713 (95.5)	277,223 (97.1)	211,433 (98.1)	22,324 (96.8)	2367 (98.1)	20,224 (98.0)	
¥**Diagnosed morbidity at age 40**	No	99,795 (98.5)	282,762 (99)	214,242 (99.4)	228,86 (99.2)	2402 (99.5)	205,29 (99.5)	<0.001
	Yes	1496 (1.5)	2715 (1.0)	1314 (0.6)	186 (0.8)	12 (0.5)	104 (0.5)	
**Country of birth**	Sweden	71,989 (71.1)	244,205 (85.5)	179,313 (83.2)	17,632 (76.4)	1615 (66.9)	17,237 (83.5)	
	Africa	1521 (1.5)	1218 (0.4)	940 (0.4)	352 (1.5)	31 (1.3)	110 (0.5)	<0.001
	Asia	7008 (6.9)	5747 (2.0)	7048 (3.3)	1272 (5.5)	230 (9.5)	708 (3.4)	
	EU without Nordics	3075 (3.0)	8840 (3.1)	8238 (3.8)	652 (2.8)	141 (5.8)	703 (3.4)	
	Europe without EU and Nordics	7093 (7.0)	6207 (2.2)	4497 (2.1)	885 (3.8)	140 (5.8)	461 (2.2)	
	North America	225 (0.2)	589 (0.2)	1605 (0.7)	65 (0.3)	17 (0.7)	87 (0.4)	
	Nordics without Sweden	8586 (8.5)	15,672 (5.5)	10,835 (5.0)	1759 (7.6)	158 (6.5)	982 (4.8)	
	Oceania	37 (<1)	74 (<1)	143 (0.1)	4 (<1)	0 (0.0)	7 (<1)	
	Russia	52 (0.1)	242 (0.1)	679 (0.3)	22 (0.1)	10 (0.4)	49 (0.2)	
	South America	1383 (1.4)	2300 (0.8)	1993 (0.9)	380 (1.6)	62 (2.6)	249 (1.2)	
	‡Unknown	322 (0.3)	383 (0.1)	265 (0.1)	49 (0.2)	10 (0.4)	40 (0.2)	
**Unemployment at age 40**	No	67307 (66.4%)	285938 (73.1%)	72832 (70.4%)	14240 (58.4%)	702 (64.8%)	21826 (81.0%)	<0.001
	Yes	7407 (7.3%)	26413 (6.7%)	4528 (4.4%)	4455 (18.3%)	159 (14.7%)	2078 (7.7%)	
	‡Missing	26577 (26.2%)	78969 (20.2%)	26053 (25.2%)	5707 (23.4%)	223 (20.6%)	3029 (11.2%)	
**Family income at age 40**	Low (0–20%)	10,359 (10.2%)	22,514 (5.8%)	4344 (4.2%)	1984 (8.1%)	108 (10.0%)	1607 (6.0%)	<0.001
	Lower middle (>20–40%)	16,102 (15.9%)	59,479 (15.2%)	11,736 (11.3%)	4135 (16.9%)	166 (15.3%)	3767 (14.0%)	
	Middle (>40–60%)	22,771 (22.5%)	71,150 (18.2%)	14,745 (14.3%)	5580 (22.9%)	242 (22.3%)	4509 (16.7%)	
	Upper middle (>60–80%)	27,700 (27.3%)	119,786 (30.6%)	19,817 (19.2%)	,7329 (30.0%)	284 (26.2%)	8278 (30.7%)	
	High (>80–100%)	19,061 (18.8%)	107,107 (27.4%)	45,817 (44.3%)	4709 (19.3%)	178 (16.4%)	8069 (30.0%)	
	‡Missing	5298 (5.2%)	11,284 (2.9%)	6954 (6.7%)	665 (2.7%)	106 (9.8%)	703 (2.6%)	
**Nr. of children at home at age 40**	0	32,587 (32.2)	126,003 (32.2%)	36,623 (35.4%)	7797 (32.0%)	364 (33.6%)	9651 (35.8%)	<0.001
	1	22,457 (22.2)	76,803 (19.6%)	16,255 (15.7%)	5196 (21.3%)	195 (18.0%)	4180 (15.5%)	
	2	32,736 (32.3)	157,864 (40.3%)	38,885 (37.6%)	8589 (35.2%)	305 (28.1%)	10,547 (39.2%)	
	3+	8213 (8.1)	19,366 (4.9%)	4696 (4.5%)	2155 (8.8%)	114 (10.5%)	1852 (6.9%)	
	‡Missing	5298 (5.2)	11,284 (2.9%)	6954 (6.7%)	665 (2.7%)	106 (9.8%)	703 (2.6%)	

Data are n (%) or p values. *p-values are reported for χ^2^ test. ‡Missing or unknown observations were included in the model as its own categories. ¥Having any of the following at age 40; mental disorders and substance use, musculoskeletal disorders, other non-communicable diseases, neurological disorders, unintentional injuries, skin diseases, chronic respiratory diseases, cardiovascular diseases, and sense organ diseases.

Table 3 shows that both men and women with high and middle educational attainment had reduced all-cause mortality, compared to those with low education. After adjusting for all covariates, women who moved from low to middle or low to high educational attainment had reduced ORs for all-cause mortality OR = 0.71 (CI: 0.66–0.77) and OR = 0.25 (CI: 0.14–0.45) compared with women with low education throughout the study period. Corresponding significant estimates were found for women moving from middle to high educational attainment as compared to having middle education throughout the study period, with 34% lower odds of mortality OR = 0.66 (CI: 0.60–0.73). The separate sibling's analysis confirms the same pattern of association as in the overall sample.

**Table 3:** Odds Ratio (OR) with 95% Confidence Intervals (CIs) for the association between highest attained education (no change, and change), in men and women between the ages 40–50 years (with low and middle education as the reference), and all-cause mortality between the ages 51–60 years (in 2011–2020).

**Men**	Highest attained education between 40 and 50 years of age							
	No change in education			Change in education		No change in education		Change in education
	*Low* (≤9 yrs)	*Middle* (10–12 yrs)	*High* (>12 yrs)	*Low to high*	*Low to middle*	*Middle* (10–12 yrs)	*High* (>12 yrs)	*Middle to high*
Crude	1	*0.73 (0.71–0.75)*	*0.40 (0.38–0.41)*	0.82 (0.66–1.0)	1.05 (0.98–1.13)	1	*0.54 (0.53–0.56)*	*0.77 (0.69–0.86)*
*Adjusted for*						1		
Birth year	1	*0.75 (0.72–0.77)*	*0.40 (0.39–0.42)*	0.83 (0.67–1.0)	1.06 (0.99–1.14)	1	*0.54 (0.52–0.56)*	*0.77 (0.68–0.86)*
¥Diagnosed morbidity	1	*0.74 (0.72–0.76)*	*0.41 (0.40–0.43)*	0.83 (0.67–1.02)	1.05 (0.98–1.23)	1	*0.55 (0.53–0.57)*	*0.78 (0.70–0.87)*
Country of birth	1	*0.74 (0.72–0.76)*	*0.41 (0.39–0.42)*	0.87 (0.71–1.08)	1.08 (1.0–1.2)	1	*0.55 (0.53–0.57)*	*0.79 (0.71–0.89)*
Nr of children at home	1	*0.68 (0.66–0.70)*	*0.38 (0.36–0.40)*	*0.67 (0.45–1.00)*	1.02 (1.00–1.09)	1	*0.56 (0.53–0.58)*	*0.65 (0.60–0.75)*
Family income	1	*0.73 (0.71–0.75)*	*0.45 (0.43–0.48)*	*0.58 (0.39–0.87)*	0.94 (0.88–1.01)	1	*0.63 (0.60–0.66)*	*0.59 (0.51–0.68)*
Unemployment	1	*0.67 (0.65–0.69)*	*0.37 (0.35–0.38)*	*0.62 (0.42–0.93)*	0.95 (0.89–1.02)	1	*0.55 (0.53–0.57)*	*0.63 (0.55–0.73)*
aAll	1	*0.75 (0.73–0.77)*	*0.49 (0.47–0.52)*	0.67 (0.45–1.01)	0.95 (0.89–1.02)	1	*0.66 (0.63–0.69)*	*0.66 (0.57–0.75)*
a‡Siblings	1	*0.81 (0.74–0.88)*	*0.55 (0.47–0.65)*	0.46 (0.11–1.94)	0.82 (0.68–1.00)	1	0.72 (0.62–0.84)	0.75 (0.48–1.16)

**Women**								
Crude	1	*0.63 (0.61–0.65)*	*0.41 (0.39–0.43)*	*0.42 (0.31–0.56)*	*0.71 (0.65–0.67)*	1	*0.65 (0.63–0.68)*	*0.68 (0.61–0.75)*
*Adjusted for*						1		
Birth year	1	*0.63 (0.61–0.66)*	*0.41 (0.40–0.43)*	*0.42 (0.32–0.57)*	*0.71 (0.07–0.77)*	1	*0.66 (0.63–0.68)*	*0.69 (0.62–0.76)*
¥Diagnosed morbidity	1	*0.63 (0.61–0.66)*	*0.42 (0.41–0.44)*	*0.42 (0.31–0.56)*	*0.70 (0.65–0.76)*	1	*0.67 (0.64–0.69)*	*0.69 (0.62–0.76)*
Country of birth	1	*0.58 (0.56–0.60)*	*0.39 (0.37–0.41)*	*0.24 (0.14–0.43)*	*0.70 (0.65–0.75)*	1	*0.67 (0.64–0.71)*	*0.63 (0.58–0.70)*
Nr of children at home	1	*0.62 (0.60–0.64)*	*0.45 (0.42–0.47)*	*0.24 (0.13–0.42)*	*0.71 (0.66–0.77)*	1	*0.72 (0.68–0.75)*	*0.64 (0.58–0.70)*
Family income	1	*0.58 (0.56–0.60)*	*0.39 (0.37–0.41)*	*0.23 (0.13–0.41)*	*0.68 (0.63–0.74)*	1	*0.67 (0.64–0.70)*	*0.63 (0.58–0.70)*
Unemployment	1	*0.61 (0.59–0.63)*	*0.45 (0.42–0.47)*	*0.25 (0.14–0.45)*	*0.71 (0.66–0.77)*	1	*0.72 (0.69–0.76)*	*0.66 (0.60–0.73)*
aAll	1	*0.70 (0.62–0.78)*	*0.52 (0.43–0.63)*	*0.10 (0.01–0.83)*	*0.75 (0.61–0.93)*	1	*0.76 (0.65–0.90)*	*0.71 (0.55–0.93)*
a‡Siblings	1	*0.58 (0.56–0.60)*	*0.39 (0.37–0.41)*	*0.24 (0.14–0.43)*	*0.70 (0.65–0.75)*	1	*0.67 (0.64–0.71)*	*0.63 (0.58–0.70)*

a Adjusted for all covariates simultaneously. ‡Those with no siblings, and sibling pairs who were concordant in the outcomes (all-cause mortality during follow-up) were removed from the analysis. ¥Having any of the following at age 40; mental disorders and substance use, musculoskeletal disorders, other non-communicable diseases, neurological disorders, unintentional injuries, skin diseases, chronic respiratory diseases, cardiovascular diseases, and sense organ diseases.

In men, those who moved from middle to high education had reduced mortality OR = 0.66 (CI:0.57–0.75). Moving from low to high or low to middle compared to having low educational attainment throughout the study period was not associated with any significant reduced risk. The same trend was seen in the sibling analyses.

For both men and women, none of the covariates, analysed one by one, seemed to drastically affect the overall effect of additional education.

## 4 Discussion

Our results show that adding years of schooling in mid-life is associated with reduced all-cause mortality in both men and women. These results are in line with previous studies showing a strong negative relationship between education and risk of all-cause mortality in both men and women (Johan et al., 2017; Ostergren, 2018; Phelan et al., 2004). To our knowledge however, this is the first study to investigate the effects on mortality of educational mobility in mid-life. Additional level of education, e.g., moving from low to middle, and from middle to high, between 40 and 50 years of age, as compared to having low education throughout the study period was beneficial for women. In men, reduced mortality was only found for those who moved from middle to high educational attainment.

The mechanisms through which adult education could relate to reduced mortality are not known. However, adult education helps strengthen and serves as a compensatory function to increase equity in the Swedish educational system. A recent report showed that those who participated in adult education improved their situation on the labour market, and that the proportion standing outside the labour market decreased. (https://vizhub.healthdata.org/gbd-compare/) The results from our study imply that a system that allows and encourages adult education, not only helps integrate people inot the labour market and society but may also have beneficial population health effects. At the same time, in Sweden, the socioeconomic background of the student is increasingly having an impact on educational outcomes (Ostergren, 2018) and hence much focus should at the same time be placed on having targeted policy intervention to address educational inequalities in youth education.

Observed gender inequalities in health returns from adult education requires further investigation. Previous studies have shown that women participating in adult education show stronger reduction in depression risks (Feinstein, 2002), higher uptake of preventive screening (Sabates & Feinstein, 2006) and higher levels of self-efficacy (Hammond and Feinstein) compared to men. No gender differences, however, were observed from Feinstein et al. (Feinstein, Hammond, Woods, & Zotero, 2003) on the impact of adult learning for a series of health behaviours (smoking, exercising, drinking) and social capital indicators (tolerance, authoritarianism, political interest). In our study, it is also possible that the reduced risk is explained in part by the age at which changes in educational status occurred, and thus could lead to changes in occupational positions and salaries. A higher proportion of women compared to men changed their educational status closer to the age of 40, while a higher proportion of men compared to women changed their educational status closer to the age of 50 (see Supplementary File Fig. 2). Thus, women had longer time to benefit from the higher education compared to men. It is also possible that women who educated themselves further were a healthier group.

### 4.1 Methodological considerations

There are some methodological issues that need to be highlighted. First, there may be some uncertainties in measuring education over time due to a revision of the Education Register in 2000 which resulted in a rise in the level of education in Sweden that year (Ludvigsson J, 2019; SCB, 2000) (Ludvigsson, Svedberg, Olén, Bruze, & Neovius, 2019). The rise in educational levels is mainly due to a revision at the upper secondary level (>9–12 years of study), and at the post-secondary level. The increase at the upper secondary level was 1,3 percent in the year 2000, compared to the 0.5 percent the year before, in 1999. Most of this increase is a result of more people being categorized with 3 rather than 2 years of upper secondary education. For post-secondary level there was an increase by 2.3 percentage, compared to 0.4 the year before (SCB, 2017; SCB https). Whether or not a certain percentage is an artefact, these changes will likely not influence our results, since the added years of schooling occurred within the categorization of educational groups that we used in our study. However, in a sensitivity analysis using altered educational classes with the post-secondary education (<3 years of education) in the ‘middle’ group, we aimed to assess a possible over-or underestimation of the effect of adult education on mortality based on our classification as well as any revisions in reporting. We found no difference in the effect of change in education on all-cause mortality after controlling for all covariates for men and women other than decreasing ORs based on shifting populations between educational classes (Supplementary File Tables 2–4).

Second, we excluded 5 percent of the population since we lacked data on education. The likely explanation is that the missing information on education is due to not finishing basic education or being an immigrant without a registered educational level. Depending on their level of education and whether they were still alive at follow-up, either an over- or underestimation of the associations may have been observed.

Third, we do not have any information on health behaviours such as smoking, physical inactivity and eating habits. It is well-known that those in lower socioeconomic groups have a higher prevalence of low physical inactivity, smoking, and higher BMI. Not accounting for these factors is a major limitation in our study, since it is possible that those who educated themselves further are a healthier group, which could explain the reduced mortality, rather than the added years of schooling. We did, however, adjust for diagnosed morbidity at age 40. This did not change the results to any important extent, although it is uncertain to what degree the national in-and out-patient register captures the occurrence of some disorders, such as mental and musculoskeletal disorders. Although results from the sibling analysis should be interpreted with some caution, these analyses intrinsically account for factors shared within families, during the participants childhood, such as for example socioeconomic factors, genetics and lifestyles.

Fourth, the cohort was restricted to those born between 1950 and 1960 to capture both shifts in educational attainment at older ages, as well as to be able to follow-up for mortality. Since majority of deaths occur after the age of 70 in Sweden, we only capture around 15 percent of deaths in the ages that we included. These results could, however, be repeated in a couple of years, to capture the outcome at older ages. A major strength with this study is that we could link the total population in our cohort by a unique identifier to educational level and additional years of schooling and eventually to deaths over a rather long period of time. Moreover, Sweden's policy of encouraging and investing resources in adult education, provided a good case study on health effects in a large enough population to assess these effects.

## 5 Conclusion

We found that additional years of schooling in mid-life was associated with reduced all-cause mortality, especially in women. In men, reduced mortality was only found for those who moved from middle to high educational attainment. The policy implication is that a system that allows and encourages additional schooling in mid-life may have important health effects, in addition to possible labour market advantages, although more thorough studies are needed to ascertain a causal effect.

**Acknowledgement**

We are grateful to Swedish Research Council for Health, Working Life and Welfare, Grant number: DNR: 2021-00176, who funded this research. The funders had no role in study design, data collection and analysis, decision to publish, or preparation of the manuscript.

## Role of the funding source

The funder of the study had no role in study design, data collection, data analysis, data interpretation, or writing of the article. The corresponding author had full access to all the data in the study and had the final responsibility for the decision to submit for publication.

## Ethical standards

The authors assert that all procedures contributing to this work comply with the ethical standards of the relevant national and institutional committees on human experimentation and with the Helsinki Declaration of 1975, as revised in 2008. The registration numbers for the ethical approvals; Dnr: 2018/1339-31/5, Dnr: 2018/2292-32, Dnr: 2019–02185, Dnr: 2021-00657.

## CRediT authorship contribution statement

**M. Balaj:** Conceptualization, Supervision, Validation, Writing – original draft, Writing – review & editing, Methodology. **H. Sjöqvist:** Conceptualization, Data curation, Formal analysis, Methodology, Software, Writing – review & editing. **L. van der Velde:** Data curation, Formal analysis, Writing – review & editing. **PA. Allebeck:** Investigation, Supervision, Validation, Writing – original draft, Writing – review & editing. **AN. Shaaban:** Data curation, Formal analysis, Writing – review & editing. **S. Swartling Peterson:** Validation, Writing – review & editing. **TA. Eikemo:** Validation, Writing – review & editing. **EE. Agardh:** Funding acquisition, Investigation, Methodology, Project administration, Resources, Software, Supervision, Validation, Writing – original draft, Writing – review & editing.

## Declaration of competing interest

None.

## Data Availability

The authors do not have permission to share data.
