# Supplementary material for: Does educational mobility in mid-life affect mortality? A cohort study covering 1.3 million individuals in Sweden
Source: SSM Popul Health. 2023 Dec 23;25:101589. doi: 10.1016/j.ssmph.2023.101589 (PMC10790084; doi:10.1016/j.ssmph.2023.101589)
Supplement: Multimedia component 1 [file mmc1.docx]

**Supplementary file**

**Figure 1.**  Flow chart.


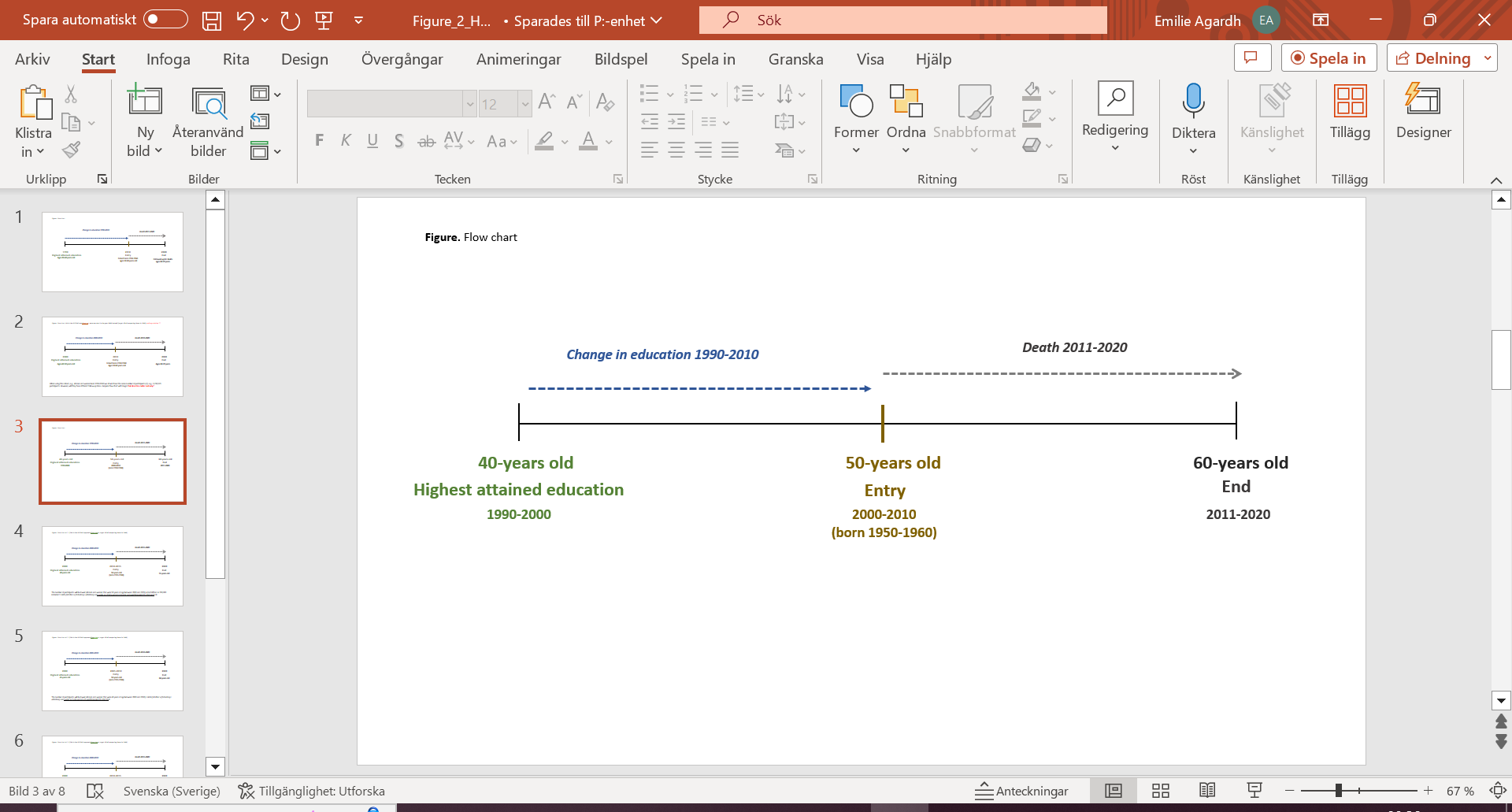


**Figure 2.** The proportion of men and women the age at which they changed their educational status.


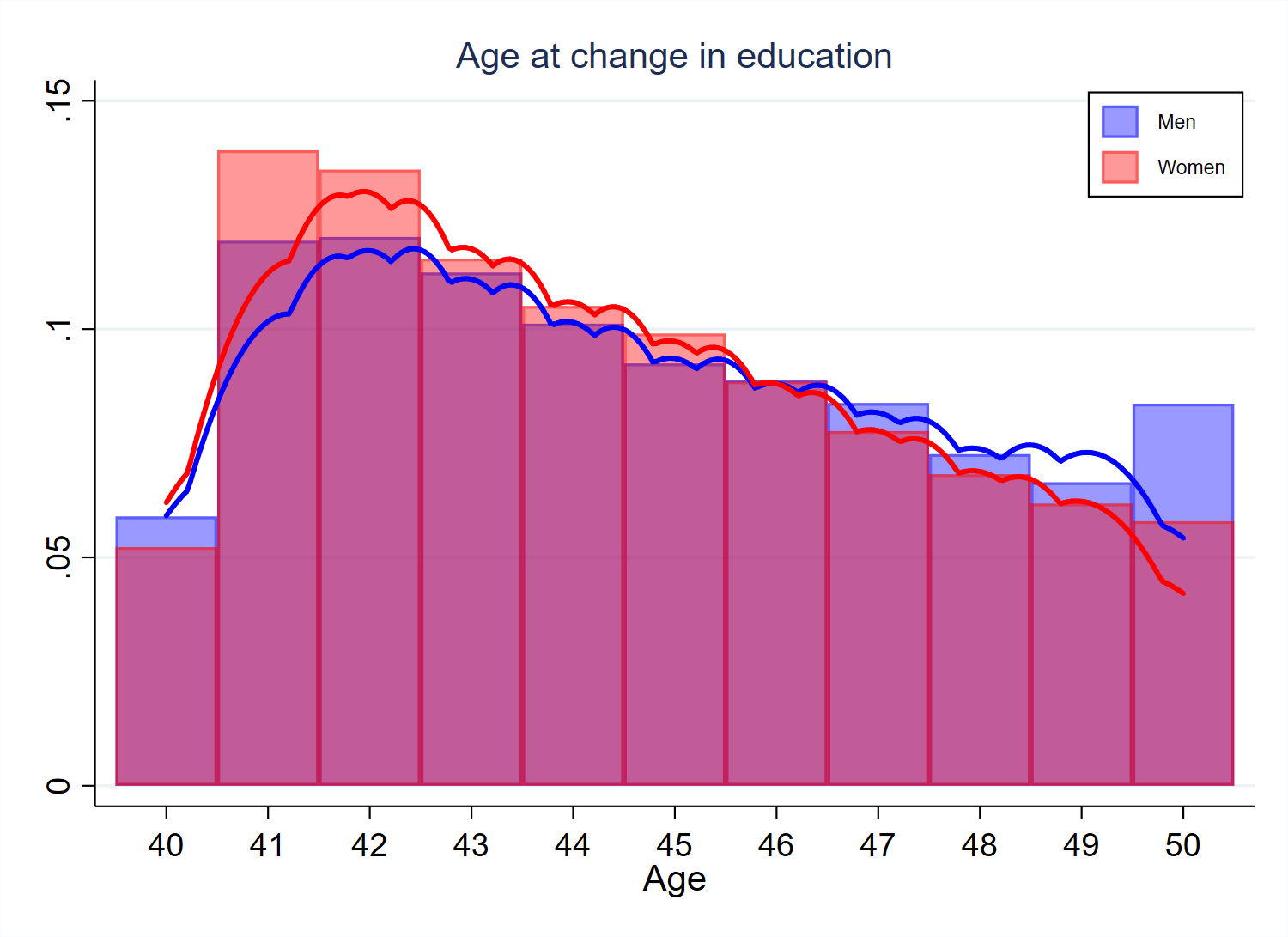


The red smoothed line: Mean proportion of women, at the age which they changed their educational status.

The blue smoothed line: Mean proportion of men, at the age which they changed their educational status.

**Table 1.** Diagnoses according to the GBD and corresponding ICD-9 and ICD-10 codes.

| **Diseases** | **ICD-9 (up to 1997)** | **ICD-10 (1998-2000)** |
| --- | --- | --- |
| Mental disorders and substance use disorders | 291-292.9, 295-295.95, 303-303.93, 304.0-304.83, 305-305.93, 307.1, 307.51, 307.54, 357.5, 760.7- 760.79, 780.59, 790.3, E850-E850.29, E850.9-E854.39, E860-E860.19 | F06.2, F10-F16.99, F18-F29.9, F50.0-F50.5, G31.2, G72.1, P04.3-P04.49, P96.1, Q86.0, R78.0-R78.5, X45- X45.9 |
| Musculoskeletal disorders | 416.1, 437.4, 446-446.9, 695.4-695.59, 710-711.99, 714-714.33, 714.8-714.9, 730.1-730.19, 732- 732.9, 733.0-733.19 | I27.1, I67.7, L93-L93.2, M00-M03.0, M03.2-M03.6, M05-M09.0, M09.2-M09.8, M30-M32.9, M34-M36.8, M40-M43.19, M65-M65.08, M71.0-M71.19, M80-M82.8, M86.3-M86.49, M87-M87.09, M88-M89.09, M89.5-M89.59, M89.7-M89.9 |
| Other non-communicable diseases | 035-035.9, 102-103.9, 133-133.6, 376.0-376.1, 680-689, 694-695.3, 707-707.9, 740-749.04, 749.2- 758.9, 759.0-759.89, 798-798.0 | A46-A46.0, A66-A67.9, B86, D86.3, H05.0-H05.119, L00-L05.92, L08-L08.9, L10-L14.0, L51-L51.9, L88- L89.95, L97-L98.499, P96.0, Q00-Q07.9, Q10.4-Q18.9, Q20-Q28.9, Q30-Q36, Q37-Q45.9, Q50-Q86, Q86.1-Q87.89, Q89-Q89.8, Q90-Q93.9, Q95-Q99.8, R95 |
| Neurological disorders | 290-290.9, 294.1-294.9, 330-331.2, 331.5-337.9, 340-341.9, 345-345.91, 349, 349.2-349.8, 353.6- 353.9, 356-356.9, 357.0-357.1, 357.3-357.4, 357.7, 358-359.9, 728.86, 728.88, 775.2 | F00-F03.91, G10-G13.8, G20-G21.0, G21.2-G24, G24.1-G25.0, G25.2-G25.3, G25.5, G25.8-G26.0, G30- G31.1, G31.8-G31.9, G35-G37.9, G40-G41.9, G61-G61.9, G70-G72, G72.2-G73.7, G90-G90.9, G95-G95.9, M33-M33.99 |
| Unintentional injuries | E850.3-E850.89, E854.8, E856-E857.09, E860.2-E869.99, E870-E876.9, E878-E879.9, E880- E886.99, E888-E906.99, E910-E928.89, E929.2-E929.5, E930-E949.9 | W00-W46.2, W49-W62.9, W64-W70.9, W73-W75.9, W77-W81.9, W83-W94.9, W97.9, W99-X06.9, X08- X32.9, X39-X39.9, X46-X47, X47.1-X47.8, X48-X48.9, X50-X54.9, X57-X58.9, Y38.9-Y84.9, Y88-Y88.3 |
| Skin diseases | 035-035.9, 102-103.9, 133-133.6, 680-689, 694-695.3, 707-707.9 | A46-A46.0, A66-A67.9, B86, D86.3, L00-L05.92, L08-L08.9, L10-L14.0, L51-L51.9, L88-L89.95, L97- L98.499 |
| Chronic respiratory diseases | 135-135.9, 136.6, 327.2-327.8, 470, 470.9-474.9, 476-476.1, 477-479, 490-504.9, 506-506.9, 508- 509, 515, 516-517.8, 518.6, 518.9, 519.1-519.4, 780.57, 786.03 | D86-D86.2, D86.89-D86.9, G47.3-G47.39, J30-J35.9, J37-J47.9, J60-J63.8, J65-J68.9, J70-J70.1, J70.8- J70.9, J82, J84-J84.9, J91-J92.9 |
| Cardiovascular diseases | 036.41-036.43, 036.6, 074.2, 074.21-074.23, 391-391.9, 392.0, 393-398.99, 402-402.91, 410-414.9, 417-417.9, 420-423, 423.1-425.9, 427-427.32, 427.6-427.89, 429.0-429.1, 430-435.9, 437.0-437.2, 437.5-437.8, 441-443.9, 447-454.9, 456, 456.3-457.9, 459, 459.1-459.39 | A39.5-A39.53, B33.2-B33.24, D86.85, G45-G46.8, I01-I01.9, I02.0, I05-I09.9, I11-I11.9, I20-I25.9, I28- I28.8, I30-I31.1, I31.8-I43.9, I47-I48.92, I51.0-I51.5, I60-I61.9, I62.0-I62.03, I63-I63.9, I65-I66.9, I67.0-I67.3 I67.5-I67.6, I68.0-I68.2, I69.0-I69.398, I70.2-I70.799, I71-I73.9, I77-I83.93, I86-I89.9, I91.9, I98 |
| Sense organ diseases | 365-365.9, 366-366.5, 366.8-366.9, 743.3, 366-366.5, 366.8-366.9, 743.3, 362.5, 367-368.9, 378-378.9, 385-385.2, 385.8-385.9, 388.1-388.2, 389-389.2, 389.7-389.9, 361-361.3, 361.8-361.9, 362-362.4, 362.6-362.9, 363-363.9, 367-, 367.5, 367.8-367.9, 368-368.6, 368.8-368.9, 369-369.4, 369.6-369.9, 377-377.7, 377.9, 378-378.9, 077-077.99, 360-360.44, 360.8-360.9, 364-364.42, 370-374.85, 374.87-376.52, 376.8-377.9, 379-380.9, 386-386.9, 388-388.9, V74.4 | H40-H40.6, H40.8-H40.9, H25-H25.2, H25.8-H25.9, H26-H26.4, H26.8-H26.9, Q12.0, H35.3, H49-H51.9, H74-H74.4, H74.8-H74.9, H90-H90.8, H91.1-H91.3, H91.8-H91.9, H94.0, H94.8, H31-H31.4, H31.8-H31.9, H33-H33.5, H33.8, H34-H34.2, H34.8-, H34.9, H35-H35.2, H35.4-H35.9, H46-H46.3, H46.8-H46.9, H47-, H47.7, H47.9, H49-H49.4, H49.8-H49.9, H50-H50.6, H50.8-H50.9, H51-H51.2, H51.8-H51.9, H52-H52.7, H53-H53.9, H54-H54.8, B30-B30.9, B31.9, B32.3, B32.4, H00-H02.8, H02.82-H02.9, H04-H05.429, H05.8-H06.3, H10-H11.9, H13-H13.8, H15-H22.8, H27-H27.9, H28.8, H32.8, H36.8, H44-H44.539, H44.8-H45.8, H48.8, H55-H55.89, H57-H58.9, H60-H62.8, H81-H83.93 |

**Table 2.** Characteristics of men aged 50 years old in 2000-2010 (born between 1950 to 1960), with highest attained education (no change, and change) between the ages 40 to 50 years (in 1990 to 2010), and who were followed-up for all-cause mortality between the ages 51-60 (in 2011 and 2020)

| **Highest attained education between 40 to 50 years of age** | | | | | | | | |
| --- | --- | --- | --- | --- | --- | --- | --- | --- |
| **Men** | | **No change in education Change in education** | | | | | | |
|  |  | *Low (*≤ 9 yrs) | *Middle (*10-15 yrs) | *High (*>15 yrs) | *Low to middle* | *Low to high* | *Middle to high* | *p-value |
| **Total** | *668,655* | 146.873 (22.0%) | 390.321 (58.3%) | 106.102 (15.9%) | 16482 (2.5%) | 630 (0.1%) | 8247 (1.2%) |  |
| **Died at age**  **51-60** | No | 138239 (94.1%) | 374675 (96.0%) | 103759 (97.8%) | 15474 (93.9%) | 605 (96.0%) | 8027 (97.3%) |  |
|  | Yes | 8634 (5.9%) | 15646 (4.0%) | 2343 (2.2%) | 1008 (6.1%) | 25 (4.0%) | 220 (2.7%) | <0.001 |
| ¥Diagnosed morbidity  **at age 40** | No | 144106 (98.1%) | 385500 (98.8%) | 105476 (99.4%) | 16158 (98.0%) | 623 (98.9%) | 8188 (99.3%) | <0.001 |
|  | Yes | 2767 (1.9%) | 4821 (1.2%) | 626 (0.6%) | 324 (2.0%) | 7 (1.1%) | 59 (0.7%) |  |
| **Country of birth** | Sweden | 120078 (81.8%) | 331849 (85.0%) | 83578 (78.8%) | 11628 (70.5%) | 341 (54.1%) | 5577 (67.6%) |  |
|  | Africa | 1565 (1.1%) | 3919 (1.0%) | 1632 (1.5%) | 485 (2.9%) | 34 (5.4%) | 251 (3.0%) | <0.001 |
|  | Asia | 5828 (4.0%) | 12235 (3.1%) | 6659 (6.3%) | 1427 (8.7%) | 111 (17.6%) | 1148 (13.9%) |  |
|  | EU without Nordics | 2854 (1.9%) | 9454 (2.4%) | 4219 (4.0%) | 411 (2.5%) | 22 (3.5%) | 303 (3.7%) |  |
|  | Europe without EU and Nordics | 5192 (3.5%) | 11393 (2.9%) | 3207 (3.0%) | 962 (5.8%) | 58 (9.2%) | 374 (4.5%) |  |
|  | North America | 267 (0.2%) | 1111 (0.3%) | 1290 (1.2%) | 94 (0.6%) | 12 (1.9%) | 101 (1.2%) |  |
|  | Nordics without Sweden | 9511 (6.5%) | 16018 (4.1%) | 3886 (3.7%) | 1035 (6.3%) | 15 (2.4%) | 234 (2.8%) |  |
|  | Oceania | 46 (<1%) | 162 (<1%) | 148 (0.1%) | 9 (0.1%) | 1 (0.2%) | 10 (0.1%) |  |
|  | Russia | 24 (<1%) | 161 (<1%) | 222 (0.2%) | 8 (<1%) | 7 (1.1%) | 8 (0.1%) |  |
|  | South America | 1115 (0.8%) | 3256 (0.8%) | 1039 (1.0%) | 326 (2.0%) | 24 (3.8%) | 190 (2.3%) |  |
|  | ‡Unknown | 393 (0.3%) | 763 (0.2%) | 222 (0.2%) | 97 (0.6%) | 5 (0.8%) | 51 (0.6%) |  |
| **Unemployment**  **at age 40** | No | 100609 (68.5%) | 212032 (70.8%) | 139225 (71.1%) | 7638 (50.0%) | 1042 (56.7%) | 5503 (59.8%) | <0.001 |
|  | Yes | 11289 (7.7%) | 28731 (9.6%) | 10364 (5.3%) | 4319 (28.3%) | 352 (19.2%) | 1349 (14.7%) |  |
|  | ‡Missing | 34975 (23.8%) | 58870 (19.6%) | 46241 (23.6%) | 3318 (21.7%) | 443 (24.1%) | 2355 (25.6%) |  |
| **Family income**  **at age 40** | Low (0-20%) | 20385 (13.9%) | 31857 (10.6%) | 11992 (6.1%) | 3099 (20.3%) | 367 (20.0%) | 1288 (14.0%) | <0.001 |
|  | Lower middle (>20-40%) | 26554 (18.1%) | 49904 (16.7%) | 17647 (9.0%) | 3110 (20.4%) | 337 (18.3%) | 1463 (15.9%) |  |
|  | Middle (>40-60%) | 29862 (20.3%) | 57009 (19.0%) | 28705 (14.7%) | 3374 (22.1%) | 371 (20.2%) | 1804 (19.6%) |  |
|  | Upper middle (>60-80%) | 43956 (29.9%) | 95369 (31.8%) | 47191 (24.1%) | 3831 (25.1%) | 392 (21.3%) | 2524 (27.4%) |  |
|  | High (>80-100%) | 21129 (14.4%) | 57500 (19.2%) | 77149 (39.4%) | 1316 (8.6%) | 183 (10.0%) | 1576 (17.1%) |  |
|  | ‡Missing | 4987 (3.4%) | 7994 (2.7%) | 13146 (6.7%) | 545 (3.6%) | 187 (10.2%) | 552 (6.0%) |  |
| **Nr. of children at home at age 40** | 0 | 67854 (46.2%) | 136500 (45.6%) | 81733 (41.7%) | 7761 (50.8%) | 834 (45.4%) | 4179 (45.4%) | <0.001 |
|  | 1 | 23934 (16.3%) | 45480 (15.2%) | 25325 (12.9%) | 2341 (15.3%) | 270 (14.7%) | 1302 (14.1%) |  |
|  | 2 | 41559 (28.3%) | 95895 (32.0%) | 67633 (34.5%) | 3595 (23.5%) | 425 (23.1%) | 2659 (28.9%) |  |
|  | 3+ | 8539 (5.8%) | 13764 (4.6%) | 7993 (4.1%) | 1033 (6.8%) | 121 (6.6%) | 515 (5.6%) |  |
|  | ‡Missing | 4987 (3.4%) | 7994 (2.7%) | 13146 (6.7%) | 545 (3.6%) | 187 (10.2%) | 552 (6.0%) |  |

Data are n (%) or p values. *p-values are reported for χ^2^ test. ‡Missing or unknown observations were included in the model as its own categories. ¥Having any of the following at age 40; mental disorders and substance use, musculoskeletal disorders, other non-communicable diseases, neurological disorders, unintentional injuries, skin diseases, chronic respiratory diseases, cardiovascular diseases, and sense organ diseases.

**Table 3.** Characteristics of women aged 50 years old in 2000-2010 (born between 1950 to 1960), with highest attained education (no change, and change) between the ages 40 to 50 years (in 1990 to 2010), and who were followed-up for all-cause mortality between the ages 51-60 (in 2011 and 2020)

| **Highest attained education between 40 to 50 years of age** | | | | | | | | |
| --- | --- | --- | --- | --- | --- | --- | --- | --- |
| **Women** | | **No change in education Change in education** | | | | | | |
|  |  | *Low (*≤ 9 yrs) | *Middle (*10-15 yrs) | *High (*>15 yrs) | *Low to middle* | *Low to high* | *Middle to high* | *p-value |
| **Total** | *648,443* | 101.291 (15.6%) | 391.320 (60.3%) | 103.413 (16,0%) | 24402 (3.8%) | 1084 (0.2%) | 26933 (4.1%) |  |
| **Died at age**  **51-60** | No | 96713 (95.5%) | 380855 (97.3%) | 101552 (98.2%) | 23619 (96.8%) | 1072 (98.9%) | 26473 (98.3%) |  |
|  | Yes | 4578 (4.5%) | 10465 (2.7%) | 1861 (1.8%) | 783 (3.2%) | 12 (1.1%) | 460 (1.7%) |  |
| ¥Diagnosed morbidity  **at age 40** | No | 99.797 (98.5%) | 387.903 (99.1%) | 102.816 (99.4%) | 1081 (99.7%) | 24207 (99.2%) | 26807 (99.5%) |  |
|  | Yes | 1494 (1.5%) | 3417 (0.9%) | 597 (0.6%) | 3 (0.3%) | 195 (0.8%) | 126 (0.5%) | <0.001 |
| **Country of birth** | Sweden | 71989 (71.1%) | 334716 (85.5%) | 83301 (80.6%) | 18511 (75.9%) | 736 (67.9%) | 22738 (84.4%) |  |
|  | Africa | 1521 (1.5%) | 1623 (0.4%) | 516 (0.5%) | 369 (1.5%) | 14 (1.3%) | 129 (0.5%) | <0.001 |
|  | Asia | 7008 (6.9%) | 8722 (2.2%) | 3861 (3.7%) | 1408 (5.8%) | 94 (8.7%) | 920 (3.4%) |  |
|  | EU without Nordics | 3075 (3.0%) | 12138 (3.1%) | 4780 (4.6%) | 734 (3.0%) | 59 (5.4%) | 863 (3.2%) |  |
|  | Europe without EU and Nordics | 7093 (7.0%) | 8011 (2.0%) | 2622 (2.5%) | 959 (3.9%) | 66 (6.1%) | 532 (2.0%) |  |
|  | North America | 225 (0.2%) | 1044 (0.3%) | 1104 (1.1%) | 69 (0.3%) | 13 (1.2%) | 133 (0.5%) |  |
|  | Nordics without Sweden | 8586 (8.5%) | 20751 (5.3%) | 5521 (5.3%) | 1854 (7.6%) | 63 (5.8%) | 1217 (4.5%) |  |
|  | Oceania | 37 (<1%) | 122 (<1%) | 96 (0.1%) | 4 (<1%) | 0 (0.0%) | 6 (<1%) |  |
|  | Russia | 52 (0.1%) | 408 (0.1%) | 481 (0.5%) | 28 (0.1%) | 4 (0.4%) | 81 (0.3%) |  |
|  | South America | 1383 (1.4%) | 3266 (0.8%) | 1005 (1.0%) | 412 (1.7%) | 30 (2.8%) | 271 (1.0%) |  |
|  | ‡Unknown | 322 (0.3%) | 519 (0.1%) | 126 (0.1%) | 54 (0.2%) | 5 (0.5%) | 43 (0.2%) |  |
| **Unemployment**  **at age 40** | No | 67307 (66.4%) | 207147 (72.6%) | 158362 (73.5%) | 13446 (58.3%) | 1496 (62.0%) | 15087 (73.1%) | <0.001 |
|  | Yes | 7407 (7.3%) | 21030 (7.4%) | 9961 (4.6%) | 4290 (18.6%) | 324 (13.4%) | 2028 (9.8%) |  |
|  | ‡Missing | 26577 (26.2%) | 57300 (20.1%) | 47233 (21.9%) | 5336 (23.1%) | 594 (24.6%) | 3518 (17.1%) |  |
| **Family income**  **at age 40** | Low (0-20%) | 10359 (10.2%) | 17573 (6.2%) | 9580 (4.4%) | 1844 (8.0%) | 248 (10.3%) | 1312 (6.4%) | <0.001 |
|  | Lower middle (>20-40%) | 16102 (15.9%) | 44959 (15.7%) | 26973 (12.5%) | 3924 (17.0%) | 377 (15.6%) | 3050 (14.8%) |  |
|  | Middle (>40-60%) | 22771 (22.5%) | 54674 (19.2%) | 32011 (14.9%) | 5344 (23.2%) | 478 (19.8%) | 3719 (18.0%) |  |
|  | Upper middle (>60-80%) | 27700 (27.3%) | 90333 (31.6%) | 51134 (23.7%) | 6984 (30.3%) | 629 (26.1%) | 6414 (31.1%) |  |
|  | High (>80-100%) | 19061 (18.8%) | 70867 (24.8%) | 84656 (39.3%) | 4450 (19.3%) | 437 (18.1%) | 5470 (26.5%) |  |
|  | ‡Missing | 5298 (5.2%) | 7071 (2.5%) | 11202 (5.2%) | 526 (2.3%) | 245 (10.1%) | 668 (3.2%) |  |
| **Nr. of children at home at age 40** | 0 | 32587 (32.2%) | 90594 (31.7%) | 74730 (34.7%) | 7414 (32.1%) | 747 (30.9%) | 6953 (33.7%) | <0.001 |
|  | 1 | 22457 (22.2%) | 58839 (20.6%) | 34896 (16.2%) | 4917 (21.3%) | 474 (19.6%) | 3503 (17.0%) |  |
|  | 2 | 32736 (32.3%) | 114676 (40.2%) | 84592 (39.2%) | 8156 (35.4%) | 738 (30.6%) | 8028 (38.9%) |  |
|  | 3+ | 8213 (8.1%) | 14297 (5.0%) | 10136 (4.7%) | 2059 (8.9%) | 210 (8.7%) | 1481 (7.2%) |  |
|  | ‡Missing | 5298 (5.2%) | 7071 (2.5%) | 11202 (5.2%) | 526 (2.3%) | 245 (10.1%) | 668 (3.2%) |  |

*Data are n (%) or p values. *p-values are reported for χ^2^ test. ‡Missing or unknown observations were included in the model as its own categories. ¥Having any of the following at age 40; mental disorders and substance use, musculoskeletal disorders, other non-communicable diseases, neurological disorders, unintentional injuries, skin diseases, chronic respiratory diseases, cardiovascular diseases, and sense organ diseases.

**Table 4.** Odds Ratio (OR) with 95% Confidence Intervals (CIs) for the association between highest attained education (no change, and change), in men and women between the ages 40 to 50 years (with low and middle education as the reference), and all-cause mortality between the ages 51 to 60 years (in 2011 to 2020).

|  | **Highest attained education between 40 to 50 years of age** | | | | | | | |
| --- | --- | --- | --- | --- | --- | --- | --- | --- |
|  | No change in education | | | Change in education | | No change in education | | Change in education |
| **Men** | *Low*  (≤9 yrs) | *Middle*  (10-15 yrs) | *High*  (>15 yrs) | *Low to high* | *Low to middle* | *Middle*  (10-15 yrs) | *High*  (>15 yrs) | *Middle to high* |
| Crude | 1 | *0.67 (0.65-0.69)* | *0.36 (0.35-0.38)* | *0.66 (0.44-0.99)* | 1.04 (0.97-1.12) | 1 | *0.54 (0.52-0.56)* | *0.66 (0.57-0.75)* |
| *Adjusted for* |  |  |  |  |  |  |  |  |
| Birth year | 1 | *0.67 (0.66-0.70)* | *0.36 (0.35-0.38)* | 0.67 (0.45-1.0) | 1.05 (0.98-1.12) | 1 | *0.54 (0.52-0.56)* | *0.67 (0.59-0.77)* |
| ¥Diagnosed morbidity | 1 | *0.68 (0.66-0.70)* | *0.37 (0.36-0.39)* | 0.68 (0.45-1.0) | 1.04 (0.97-1.11) | 1 | *0.55 (0.53-0.57)* | *0.66 (0.58-0.76)* |
| Country of birth | 1 | *0.73 (0.72-0.76)* | *0.41 (0.40-0.43)* | 0.82 (0.67-1.02) | 1.03 (0.96-1.10) | 1 | *0.56 (0.54-0.58)* | *0.77 (0.69-0.87)* |
| Nr of children at home | 1 | *0.78 (0.75-0.80)* | *0.48 (0.47-0.50)* | *0.75 (0.60-0.93)* | 0.95 (0.89-1.02) | 1 | *0.62 (0.60-0.65)* | *0.74 (0.66-0.82)* |
| Family income | 1 | *0.73 (0.71-0.75)* | *0.40 (0.39-0.42)* | *0.77 (0.62-0.96)* | 0.96 (0.90-1.03) | 1 | *0.55 (0.53-0.57)* | *0.74 (0.66-0.83)* |
| Unemployment | 1 | *0.80 (0.77-0.82)* | *0.52 (0.50-0.54)* | 0.84 (0.67-1.04) | 0.96 (0.89-1.03) | 1 | *0.65 (0.63-0.68)* | *0.77 (0.69-0.87)* |
| *All | 1 | *0.82 (0.75-0.89)* | *0.59 (0.52-0.67)* | 1.09 (0.54-2.23) | *0.79 (0.65-0.96)* | 1 | *0.74 (0.66-0.84)* | 0.75 (0.50-1.11) |
| *‡Siblings | 1 | *0.73 (0.72-0.76)* | *0.41 (0.40-0.43)* | 0.82 (0.67-1.02) | 1.03 (0.96-1.10) | 1 | *0.56 (0.54-0.58)* | *0.77 (0.69-0.87)* |
|  |  |  |  |  |  |  |  |  |
| **Women** |  |  |  |  |  |  |  |  |
| Crude | 1 | *0.58 (0.56-0.60)* | *0.39 (0.37-0.41)* | *0.24 (0.13-0.42)* | *0.70 (0.65-0.76)* | 1 | *0.67 (0.63-0.70)* | *0.63 (0.58-0.69)* |
| *Adjusted for* |  |  |  |  |  |  |  |  |
| Birth year | 1 | *0.58 (0.56-0.61)* | *0.39 (0.37-0.41)* | *0.24 (0.14-0.42)* | *0.70 (0.65-0.76)* | 1 | *0.67 (0.63-0.70)* | *0.64 (0.59-0.71)* |
| ¥Diagnosed morbidity | 1 | *0.59 (0.57-0.61)* | *0.40 (0.38-0.42)* | *0.24 (0.14-0.43)* | *0.71 (0.66-0.77)* |  | *0.67 (0.64-0.71)* | *0.64 (0.58-0.70)* |
| Country of birth | 1 | *0.55 (0.53-0.57)* | *0.37 (0.35-0.39)* | *0.24 (0.14 (0.42)* | *0.69 (0.63-0.74)* | 1 | *0.67 (0.64-71)* | *0.64 (0.58-0.70)* |
| Nr of children at home | 1 | *0.58 (0.56-0.60)* | *0.39 (0.37-0.41)* | *0.24 (0.13-0.42)* | *0.70 (0.65-0.76)* | 1 | *0.67 (0.63-0.70)* | *0.63 (0.58-0.70)* |
| Family income | 1 | *0.63 (0.61-0.65)* | *0.46 (0.44-0.49)* | *0.24 (0.13-0.42)* | *0.69 (0.64-0.75)* | 1 | *0.73 (0.69-0.77)* | *0.64 (0.58-0.70)* |
| Unemployment | 1 | *0.58 (0.56-0.60)* | *0.39 (0.37-0.42)* | *0.23 (0.13-0.41)* | *0.68 (0.63-0.74)* | 1 | *0.68 (0.65-0.72)* | *0.62 (0.56-0.68)* |
| *All | 1 | *0.61 (0.59-0.63)* | *0.46 (0.44-0.49)* | *0.25 (0.14-0.45)* | *0.70 (0.64-0.75)* | 1 | *0.74 (0.71-0.78)* | *0.67 (0.61-0.73)* |
| *‡Siblings | 1 | *0.70 (0.62-0.78)* | *0.54 (0.45-0.65)* | *0.09 (0.01-0.75)* | *0.76 (0.61-0.93)* | 1 | *0.77 (0.66-0.91)* | *0.71 (0.54-0.92)* |

¥Having any of the following at age 40; mental disorders and substance use, musculoskeletal disorders, other non-communicable diseases, neurological disorders, unintentional injuries, skin diseases, chronic respiratory diseases, cardiovascular diseases, and sense organ diseases. *Adjusted for all covariates simultaneously. ‡Those with no siblings, and sibling pairs who were concordant in the outcomes (all-cause mortality during follow-up) were removed from the analysis.
